# Accelerated Direct Carbonation of Steel Slag and Cement Kiln Dust: An Industrial Symbiosis Strategy Applied in the Bergamo–Brescia Area

**DOI:** 10.3390/ma16114055

**Published:** 2023-05-29

**Authors:** Giada Biava, Annalisa Zacco, Alessandra Zanoletti, Giampiero Pasquale Sorrentino, Claudia Capone, Antonio Princigallo, Laura Eleonora Depero, Elza Bontempi

**Affiliations:** 1 INSTM and Chemistry for Technology Laboratory, Department of Mechanical and Industrial Engineering, University of Brescia, Via Branze 38, 25123 Brescia, Italy; g.biava@unibs.it (G.B.); annalisa.zacco@unibs.it (A.Z.); alessandra.zanoletti@unibs.it (A.Z.); g.sorrentino002@unibs.it (G.P.S.); laura.depero@unibs.it (L.E.D.); 2 Italcementi-Heidelberg Materials, Via Stezzano, 87, 24126 Bergamo, Italy; c.capone@italcementi.it (C.C.); a.princigallo@italcementi.it (A.P.)

**Keywords:** aqueous carbonation, electric arc furnace (EAF) slag, argon oxygen decarburization (AOD) slag, ladle furnace (LF) slag, cement kiln dust (CKD), fly ash

## Abstract

The carbonation of alkaline industrial wastes is a pressing issue that is aimed at reducing CO_2_ emissions while promoting a circular economy. In this study, we explored the direct aqueous carbonation of steel slag and cement kiln dust in a newly developed pressurized reactor that operated at 15 bar. The goal was to identify the optimal reaction conditions and the most promising by-products that can be reused in their carbonated form, particularly in the construction industry. We proposed a novel, synergistic strategy for managing industrial waste and reducing the use of virgin raw materials among industries located in Lombardy, Italy, specifically Bergamo–Brescia. Our initial findings are highly promising, with argon oxygen decarburization (AOD) slag and black slag (sample 3) producing the best results (70 g CO_2_/kg slag and 76 g CO_2_/kg slag, respectively) compared with the other samples. Cement kiln dust (CKD) yielded 48 g CO_2_/kg CKD. We showed that the high concentration of CaO in the waste facilitated carbonation, while the presence of Fe compounds in large amounts caused the material to be less soluble in water, affecting the homogeneity of the slurry.

## 1. Introduction

Human industrial activities have led to the emissions of long-lived greenhouse gases, such as carbon dioxide (CO_2_), methane (CH_4_), nitrous oxide (N_2_O), halocarbons (R-X), and ozone (O_3_). Among these, CO_2_ is responsible for about two-thirds of the enhanced greenhouse effects that have resulted in extreme weather and environmental disasters, such as floods, droughts, and sea level rise [1]. The latest report from the Intergovernmental Panel on Climate Change (IPCC) in 2022 indicates that global net anthropogenic greenhouse gas emissions were 59 ± 6.6 GtCO_2_-eq in 2019, which is about 12% higher than in 2010 and 54% higher than in 1990 [2]. Unfortunately, the global energy demand is expected to increase by 28.6% by 2040 compared with 2017, and the transition from fossil fuels to renewable energies is not happening as quickly as necessary. Therefore, technologies that capture, utilize, and store CO_2_ (CCUS) are critical to reducing GHG emissions. It is estimated that CCUS can capture more than 7 billion tons of CO_2_ annually by 2050. CO_2_ storage options include geological, oceanic, and mineral storage. However, geological and oceanic storage technologies have potential risks, such as geological deformation, acidification of underground water or seawater, destruction of marine ecosystems, or increased earthquake frequency [1]. The European Union has defined terms and conditions for the storage of CO_2_ in legislation 2009/31/EC [3]. Mineral storage, also known as mineral carbonation, is a safer and more efficient alternative to the other mentioned technologies in situations where geological CO_2_ storage is feasible [4,5]. Mineral carbonation mimics the natural weathering process of rocks in which calcium (Ca) and magnesium (Mg) react with CO_2_ to form stable carbonates (CaCO_3_ and MgCO_3_). This process is thermodynamically favored and exergonic, meaning it releases energy spontaneously. An alkaline pH favors the dissolution of CO_2_ into materials and the production of carbonate ions (CO_3_^2−^) [6,7].

Accelerated carbonation can use a variety of starting materials, including natural minerals, such as wollastonite, serpentine, and forsterite, as well as industrial waste, such as alkaline ashes, municipal solid waste ashes, cement kiln dust, cement-based materials, pulp/paper mill wastes, and steel slags [1,8]. Using industrial alkaline wastes for carbonation offers several potential advantages, including reduced energy consumption and costs due to the exothermic reaction; stable products that can be reused in construction materials; efficient sequestration at milder conditions; and mitigation of environmental impacts through the stabilization of heavy metals trace elements, such as Pb, Ni, and Cd [9,10]. However, the disadvantages of this approach include the low abundance of industrial alkaline wastes in nature compared with minerals, and their potential for carbon dioxide sequestration is not as great as that of alkaline minerals (contains Ca or Mg) or materials designed for the CO_2_ capture [11,12].

Steel slags are a particularly interesting by-product for carbonation given that steel industries are major producers of CO_2_, together with cement plants (respectively 6% and 7% of global CO_2_ emissions in 2020) [13]. The European crude steel production reached 152 Mt in 2021 [14], whereas the Italian production was 24.4 Mt/year in 2021. In Italy alone, 1.8 million tons of steel slags were produced in 2017 [15]. Due to their widespread availability and chemical and mineralogical composition, steel slags are particularly well-suited for mineral carbonation [10]. Steel slags are classified based on their production process, including basic oxygen furnace (BOF) steel slag, electric arc furnace (EAF) steel slag, and ladle furnace (LF) steel slag. These by-products contain a mixture of many compounds, primarily calcium, iron, silicon, magnesium, aluminum, and manganese oxides, which exist in different mineralogical phases [16]. Fresh steel slags typically contain three main calcium phases: portlandite (Ca(OH)_2_), Ca-(Fe)-silicate (e.g., hedenbergite (CaFeSi_2_O_6_), garnet (Ca_1_._92_Fe_3_._08_O_12_Si_3_)), and Ca-Fe-O (e.g., brownmillerite (Ca_2_(Al,Fe^3+^)_2_O_5_), as well as several mineral phases, including Mg-Fe-O (e.g., magnesioferrite (MgFe_2_O_4_)), Fe-O (e.g., wuestite (FeO) and magnetite (Fe_3_O_4_)), and trace amounts of calcite (CaCO_3_) [17,18,19,20,21]. Due to their high content of basic oxides, steel slags are highly alkaline (pH~12), and their total theoretical CO_2_ sequestration capacity was evaluated at approximately 0.13–0.25 kg of CO_2_/kg of slag based on the total calcium content [16,22].

The direct aqueous process was identified as the most effective method for the carbonation of industrial waste. This process is carried out in one step and involves the simultaneous dissolution of reactive species, such as Ca^2+^ and Mg^2+^ ions, and the precipitation of carbonate products and can be carried out in two ways. For waste materials with a high silicate content, the slurry phase method is employed at a liquid-to-solid ratio of 5–50 L/kg, while the thin-film route method is used for L/S ratios <1.5 L/kg [10]. The aqueous carbonation mechanism involves several reactions [1]:

(Ca, Mg)_x_Si_y_O_x+2y+z_H_2z_(s) + xCO_2_ (g) → x(Ca/Mg)CO_3_ (s) +ySiO_2_ (s) + zH_2_O (g/l) (1)

CO_2_ (g) + H_2_O (l) → H_2_CO_3_ (aq) (2)

H_2_CO_3_ (aq) → H^+^ + HCO_3_^−^ (3)

HCO_3_^−^ → H^+^ + CO_3_^2−^ (4)

Over the last decade, several studies have investigated the direct aqueous carbonation process, which has shown promising results. However, due to the different routes used, comparing the studies has been challenging. Factors such as pH, liquid-to-solid ratio, pressure, temperature, and starting material properties (composition, particle size, porosity, and surface area) influence the process and must be balanced to achieve efficient CO_2_ sequestration [23,24].

The primary objective of this research was to assess the carbonation potential of various industrial wastes produced in the Bergamo–Brescia region of Italy and identify the most viable ones for CO_2_ sequestration through an accelerated aqueous carbonation process. To make the reactions comparable, specific parameters were selected. After a thorough examination of the industrial landscape of the area, this study focused on steel slag and cement by-products, as Brescia accounts for 52% of Italian steel production and 36% of the Lombardy region’s steel production [14], while Bergamo is significant for cement production activities.

The goal of the project was to capture CO_2_ simultaneously from the flue gas emissions of steel and cement plants and carbonate industrial wastes to create new resources for low-carbon construction materials. Cement kiln dust can be used in cement bricks, concrete, or mortar as cementitious materials [25], and steel slag can serve various purposes, depending on its pozzolanic and hydration activity and f-CaO/MgO ratio, such as a supplementary cementitious material (SCM), pure binder material with the addition of carbonation treatment, aggregates, concrete blocks, and self-healing concrete [24,26,27,28]. This approach enables multiple industries located in the same area to collaborate and form a network that focuses on a circular economy, following the principles of industrial symbiosis.

## 2. Materials and Methods

### 2.1. Starting Industrial By-Products

This study focused on collecting two types of industrial by-products: cement kiln dust (CKD) and steel slags.

Cement kiln dust is a fine-grained alkali-rich dust, and it is a secondary material derived from air pollution control devices during cement clinker production. CKD is composed of partially calcined or unreacted raw feed, clinker dust, fuel combustion ashes, alkali compounds, halides, and some trace elements. Its composition varies depending on factors such as the type of fuel used and the composition of the raw feed, the type of cement production operation, the dust collection facility, and the typology of fuel used [28,29,30].

On the other hand, steel slags were collected from three different steel plants located in the Brescia province. The samples included electric arc furnace (EAF) slag, ladle furnace (LF) slag, and argon oxygen decarburization (AOD) slag. EAF slag is formed from the oxidation of the scrap and additives inserted in the electric furnace charge, while LF slag results from refining outside the furnace in the ladle [31]. AOD slag belongs to the LF slag group and is produced during the argon oxygen decarburization process [32]. The chemical and physical properties of steel slags depend on their composition, which includes calcium oxide (CaO), silicon dioxide (SiO_2_), iron oxide (FeO), and other compounds.

### 2.2. Sample Pre-Treatment

Steel slags typically have a particle distribution of 1–5 cm, but research showed that smaller particles have a greater reactivity in the carbonation process [8,23,33]. Therefore, before use in carbonation reactions, steel slags were first dried at 105 °C for 2 h to remove any moisture, and then crushed and ground using a crusher (Herzog HSM-100P) and a disc mill (Retsch BB51-WC) to obtain a fine powder using a tungsten jar for 2 min.

For the EAF_3 sample, silica fume (SiO_2_) at 20% in weight and MilliQ water (200 mL) were added to the sample to increase its erosion resistance prior to the carbonation reaction using a rotary shaker. After 2 weeks, the mix was dried at 105 °C overnight and then crushed using a jar mill (Retsch MM400). EAF_2 was crushed in two different ways to test the efficiency of the silica fume in the carbonation process: one method involved crushing using a disc mill, while the other involved erosion with silica (50% in weight) for increased hardness compared with EAF_3, following the same process as for EAF_3.

In contrast, the cement kiln dust did not require any pre-treatment, as it was already in powder form. All samples were sieved (Ø = 106 μm) to ensure the homogenization of the particles.

### 2.3. Characterization

#### 2.3.1. XRD Analysis

To identify the crystalline components of the starting materials and carbonated products, X-ray diffraction analysis (XRD) was performed using a PANalytical X’Pert PRO diffractometer. The instrument was equipped with a Cu Kα anode and operated at 40 kV and a current of 40 mA, with the scans (2θ) having a step interval of 0.017°. The mineralogical composition was determined using the instrumental software PANalytical X’Pert HighScore Plus version 2.1.0, which was connected to the ICDD PDF2 database (1998).

Quantitative XRD analyses were carried out through the Rietveld method using the open-source software PROFEX (version 5.0.2, released on 22 September 2022) [34], as described in a previous study [35]. The internal standard used was corundum (Al_2_O_3_), which was present at a weight percentage of 25%, as it was not found in the analyzed samples. Reference mineralogical phases were obtained from the Crystallography Open Database (COD). This method enabled the calculation of the amount of calcium carbonate and, consequently, the amount of CO_2_ sequestered. Before the analysis, the samples were homogenized and crystals of similar size were obtained via sieving (Ø = 90 μm).

#### 2.3.2. XRF Analysis

To determine the chemical composition of the starting material, a Bruker S8 Tiger 4 kW spectrophotometer was employed. This instrument used X-rays from an Rh tube and a mixture of argon (90%) and methane (10%) as the detector gas. Prior to the analysis, the sample was compressed into a bead and heated to 950 °C to eliminate any moisture and carbon dioxide. A total of 1.20 g of the calcined sample was then added to 12 g of flux consisting of 70% lithium tetraborate and 30% lithium metaborate. At a temperature of 1100 °C, this flux acted as a solvent, dissolving the oxide components. Prior to the analysis, the samples were sieved (Ø = 90 μm) to ensure a homogeneous sample and crystals of similar size.

#### 2.3.3. SEM Analysis

The morphology of the selected starting material and the corresponding carbonated products (AOD_1, LF_2, EAF_3 + SiO_2_, CKD) was analyzed using a ZEISS EVO MA-15 instrument. The instrument was equipped with a LaB-6 filament as the electron source, and the analysis was performed using secondary electron imaging. Prior to the analysis, the samples were coated with Pd particles to enhance the conductivity, and the chemical composition was identified using AZTEC software version 6.1.7601 (Oxford Instruments, Abingdon, UK).

#### 2.3.4. IR Analysis

The Nicolet iN10 Infrared Microscope (ThermoFisher Scientific, Milan, Italy) was used to perform FT-IR analysis on the selected starting materials and corresponding carbonated products (AOD_1, LF_2, EAF_3 + SiO_2_, CKD) in transmittance mode using a barium fluoride (BaF_2_) window. Each spectrum was collected with a spectral resolution of 8 cm^−1^ over 16 scans. The obtained spectra were analyzed using OMNIC software version 9.11.475 (ThermoFisher Scientific, Milan, Italy) to interpret the results.

### 2.4. Accelerated Carbonation Test

**Figure 1:**
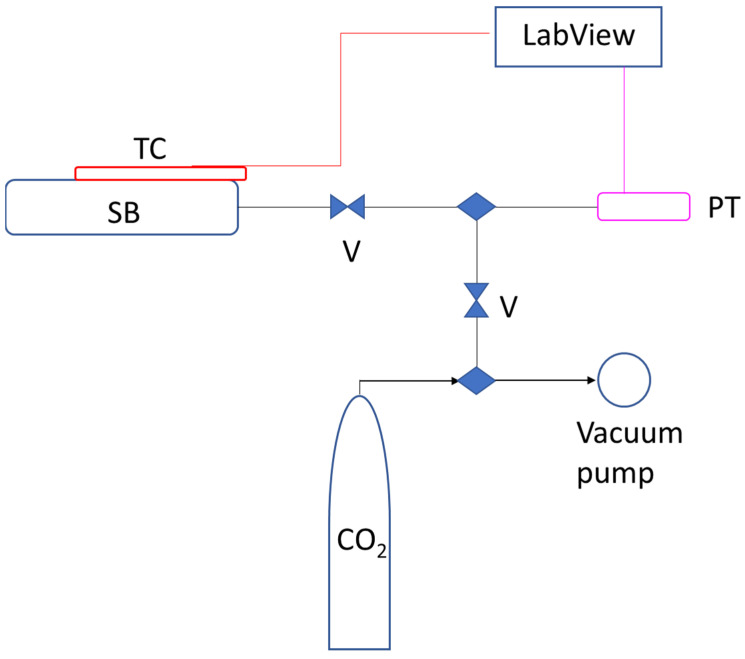
Scheme of the experimental setup: sample barrel (SB), thermocouple (TC), needle valves (V), and pressure transducer (PT).

The experimental setup used for the accelerated carbonation was described in detail in a recent publication [6] and is illustrated in Figure 1. The setup was constructed of durable, stainless steel components made from either AISI 304 or 316L. The sample was manually loaded into a 150 mL volume sample barrel (SB) using a pipette and then connected via a needle valve (V) to a precision pressure transducer (PT), which continuously recorded gas pressure values with an accuracy of 0.5%. These pressure values were collected at a rate of 100 samples per second. The temperature outside the barrel was monitored using a thermocouple (TC) placed on the outer wall of the barrel. Pressure and temperature data were collected using LabVIEW data acquisition software and were recorded every 5 s. Before the carbonation process began, the barrel was evacuated of excess air using a vacuum pump. Once the CO_2_ injection began and the pressure reached 15 bar, the valve was closed to isolate the system. Throughout the entire process, including preparation, loading, and carbonation, the loaded sample, CO_2_, and final setup were weighed using an electronic scale with an accuracy of 0.1 g. The barrel and the circuit were weighed before and after carbonation to ensure the system remained isolated. If the difference between the total initial mass and the final mass was zero, the system was confirmed as closed. Once the carbonation reaction was complete, the setup was disassembled, and each component was cleaned with acetone to remove all solid or liquid impurities. Prior to conducting the tests, a leak test was performed by charging the circuit with helium at a pressure of approximately 20 bar and room temperature for 48 h.

The accelerated carbonation tests followed the thin-film route, with a liquid-to-solid ratio (L/S) of 1.5 L/kg. To prepare the sample, approximately 30 g of material was mixed with Milli-Q water (Millipore DirectQ-5 TM, Millipore S.A.S, 67,120, Molsheim, France) for 10 min to ensure a homogeneous slurry. The experiments were carried out at room temperature with a CO_2_ pressure of 15 bar (99.99% purity, supplied by Sol) and lasted for 24 h. The slurry was then extracted and dried at 105 °C for 24 h to remove any remaining water before the XRD and SEM analysis. The amount of CO_2_ sequestered was calculated using three different methods:

1. The Rietveld method with an internal standard was used to calculate the amount of sequestered CO_2_. This was done by comparing the weight percentage of calcium carbonate before and after carbonation and considering the stoichiometric ratio between CaCO_3_ and CO_2_ in the following reactions:

Ca_2_SiO_4_ + 2CO_2_ → 2CaCO_3_ + SiO_2_ (5)

Ca(OH)_2_ + CO_2_ → CaCO_3_ + H_2_O (6)

2. The amount of CO_2_ sequestered was calculated using the difference between the final mass of the CO_2_ sequestered and the initial mass of CO_2_ injected. The system was closed, and the components of the setup, inserted slurry, and CO_2_ were weighed at each step of the test. The mass of CO_2_ sequestered was the difference between the final mass of the setup after removing the unreacted CO_2_ and the initial mass of the setup. If the final mass and the initial mass of the setup corresponded, there was no loss of CO_2_.
3. The perfect gas law was applied by monitoring the pressure during the entire process using the initial pressure (p_i_), final pressure (p_f_), and initial mass of CO_2_, as determined via the following equation:

m_f_(CO_2_) sequestered = m_i_ (CO_2_) * (1 – p_f_/p_i_) (7)

## 3. Results and Discussion

**Figure 2:**
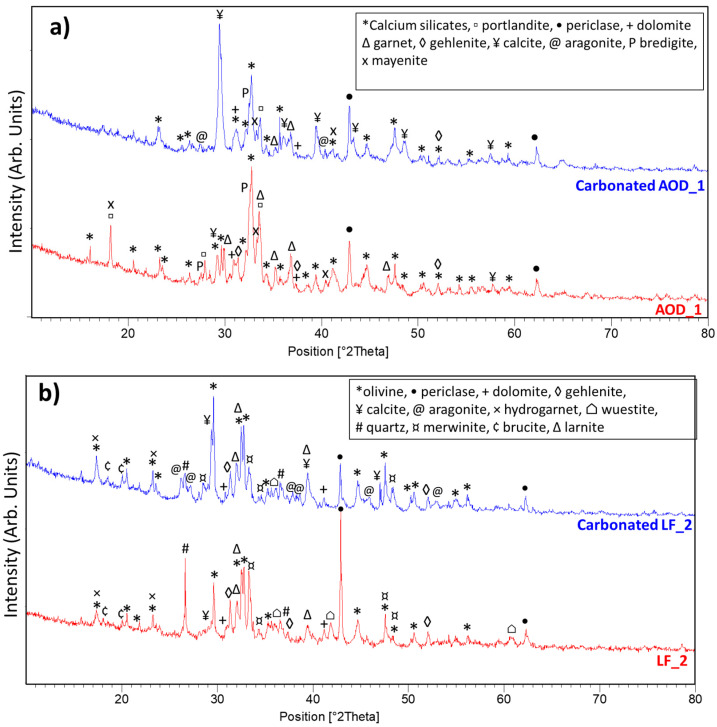

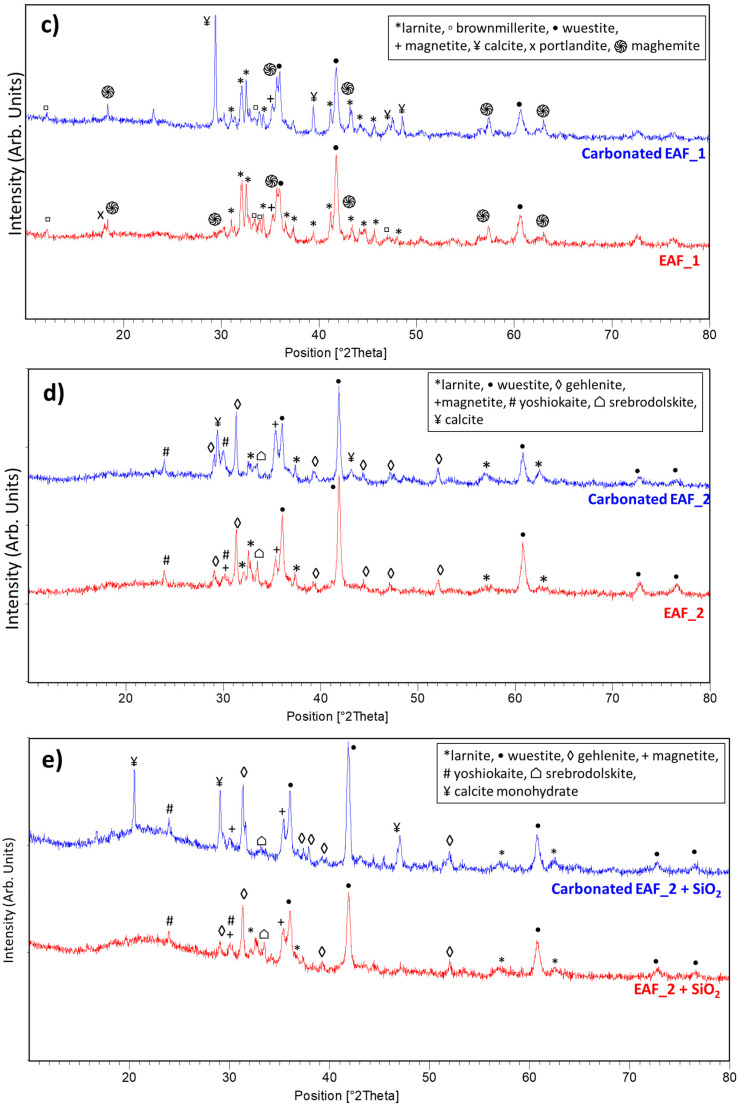

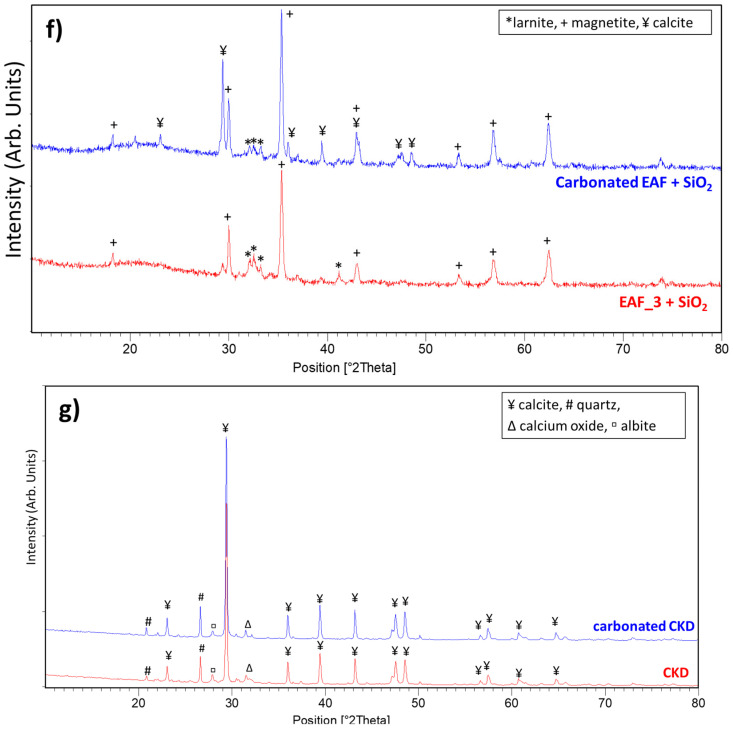
XRD spectra of steel slags and related carbonated products: (**a**) AOD_1 and carbonated AOD_1; (**b**) LF_2 and carbonated LF_2; (**c**) EAF_1 and carbonated EAF_1; (**d**) EAF_2 and carbonated EAF_2; (**e**) EAF_2 + SiO_2_ and carbonated EAF_2 + SiO_2_; (**f**) EAF_3 + SiO_2_ and carbonated EAF_3 + SiO_2_; (**g**) CKD and carbonated CKD. The reference codes of the mineralogical phases are presented in Table A1.

Table 1 presents the chemical composition results of the selected wastes, which were highly variable depending on the type of steel scraps and process [26]. LF/AOD slags contained higher amounts of reactive species, such as CaO and MgO, while EAF slags had a higher percentage of Fe_2_O_3_. These findings are consistent with literature reports [11,26,30,31,36,37,38]. Notably, the EAF_3 sample had a higher percentage of SiO_2_ due to the use of SiO_2_ fume in the grinding process. CKD had a high CaO content (44.7%), but the loss on ignition (LOI) of 29.92 must be taken into account, which represented the weight loss of the sample due to the decarbonation process. As suggested by Abdel-Ghani et al. [28], CKD was partially calcined, and the loss of weight was likely due to the loss of CO_2_ as a result of the presence of calcite (CaCO_3_) as the main phase in the XRD spectrum shown in Figure 2g, which is consistent with previous reports [11,30,38].

The XRD analysis results of the steel slags and carbonated products are presented in Figure 2. The AOD_1 slag and LF_2 slag had similar main phases, including periclase (MgO), gehlenite (Ca_2_Al(AlSi)O_7_), and dicalcium silicate. The presence of silicates was also found in the IR spectrum (Figures S4 and S5 in Supplementary Materials): silicates have vibrations in the region of 1200–800 cm^−1^ due to symmetric stretching vibrations of Si-O bonds. Peaks in this range are given by β-C_2_S, γ-C_2_S, merwinite, and bredigite [39]. For the EAF_1 slag, the main phases were larnite (Ca_2_SiO_4_), wuestite (FeO), maghemite (Fe_2_O_3_), and brownmillerite (4CaO·Al_2_O_3_·Fe_2_O_3_). The main phases for EAF_2 were gehlenite (Ca_2_Al(AlSi)O_7_), wuestite (FeO), magnetite (Fe_3_O_4_), and larnite (Ca_2_SiO_4_), as confirmed by the XRD patterns in Figure 2c,d. These two last phases were the main constituent for EAF_3, as shown in Figure 2e. For this last material, IR analysis confirmed the identified mineralogical phases: β-C_2_S has a vibration band at 805 cm^−1^, amorphous silica has a stretching bond at 1111 cm^−1^, and the carbonate ion has a bending vibration at 871 cm^−1^ and a stretching vibration at 1469 cm^−1^ (see the Supplementary Materials, Figure S6). These findings are consistent with previously reported data in the literature [23,26,40,41].

The accelerated carbonation tests were carried out under uniform reaction conditions (p = 15 bar, room temperature, and L/S = 1.5 L/kg, with the particle size less than 106 µm), as described in the previous section. Upon reaching 15 bar pressure, the CO_2_ valve was closed and the initial mass of CO_2_ injected into the reactor was determined using an electronic scale, which was approximately 2.50–2.60 g depending on environmental factors. The pH of the slurry was measured using litmus paper before (pH_i_) and after (pH_f_) the carbonation. The initial (p_i_) and final pressure (p_f_) data are summarized in Table 2.

**Figure 3:**
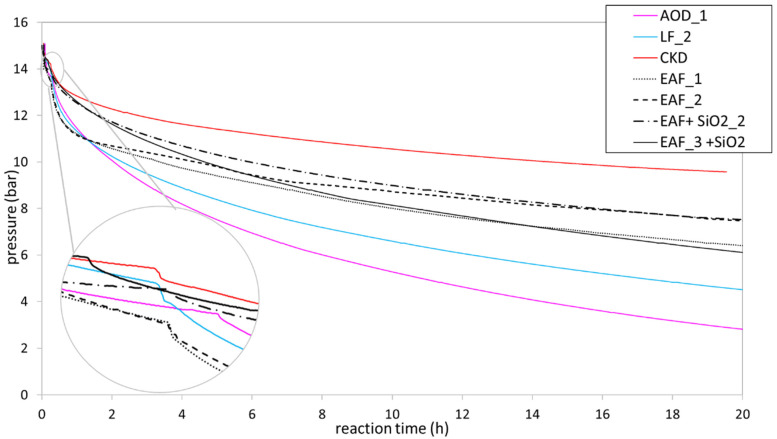
Pressure trend over 20 h for all investigated samples during the carbonation tests.

The pressure trend during the reaction was studied by collecting data at different time intervals. Figure 3 presents the pressure data collected for a reaction time of 20 h. All data were collected for 24 h, except for CKD, which was limited to 20 h due to external factors (the data present in Table 2 is related to the pressure after 24 h). The pressure decreased in all cases but in different ways due to the reactivity of the starting material. Generally, after 24 h, the pressure tended to decrease, slowly stabilizing at the values reported in Table 2. In the cases of AOD and LF, the pressure value was low, indicating the nearly complete reaction of CO_2_, whereas, for EAF and CKD, the values were higher, probably due to the absence of free Ca ions in the solution or a slower reaction rate between the slurry and the gas. It is worth noting that a small “jump” in the pressure trend was observed in the grey circle. This was due to the change in the position of the setup from a vertical to a horizontal orientation, which increased the contact surface between the slurry and the gas.

**Figure 4:**
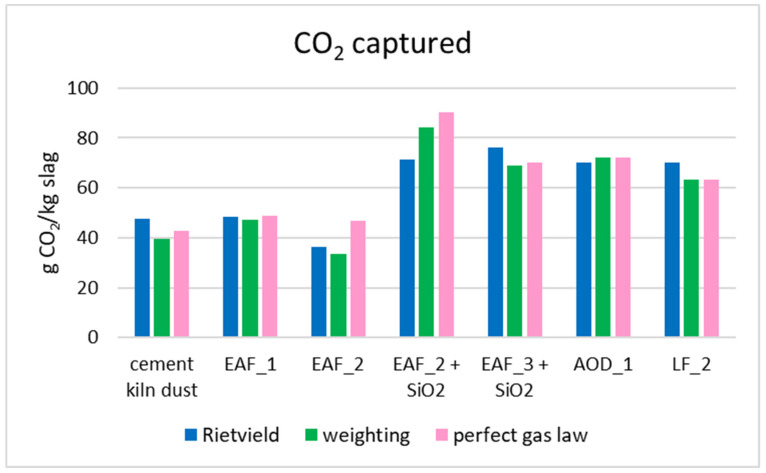
CO_2_ captured during the accelerated carbonation tests, as calculated using three different methods.

The Rietveld method is commonly used to calculate the amount of captured CO_2_. However, due to the complex XRD spectra of slags, which are characterized by a high background (fluorescence effect of iron) in EAF slags and many mineralogical phases not being well separated in LF/AOD slags, three methods were used to calculate the CO_2_ sequestered, as described in Section 2.4 The data about the amount of CO_2_ sequestered are presented in Figure 4. While the three methods showed a correlation in results, some differences were observed. For instance, EAF_2 contained a high percentage of Fe_2_O_3_, which can cause rapid separation of the slurry. During the sample charging process, the mass of water could be larger, leading to CO_2_ being only diluted in water but not adsorbed by the sample, resulting in an overestimation of CO_2_ sequestered. Moreover, for CKD, EAF_3, and LF_2, the overestimation of CO_2_ sequestered by the Rietveld method may have been due to the lack of identification of matching mineralogical phases.

When comparing the CO_2_ sequestration capacity of steel slags, it appears that the AOD and LF slags showed similar performances, which was likely due to their specific mineralogical and chemical composition. As shown in Table 1 and Figure 2a,b, these slags were rich in Ca species, such as dicalcium silicates (from 20.7% to 12.3% for AOD_1 and from 13.1% to 8.3% for LF_2), merwinite (from 4.6% to 0.5% for LF_2), gehlenite (from 8.1% to 5.4% for LF_2), and mayenite (from 12% to 2.8% for AOD_1), which are reactive in the carbonation process [42,43]. In fact, the concentrations of these phases decreased, as shown in Table A2 and the XRD patterns (Figure 2a,b). This trend was also confirmed by Johnson et al. [44] This evidence can also explain the pressure trend of AOD_1 and LF_2 in Figure 3. In fact, the presence of different Ca species enhanced the leaching of a major quantity of Ca^2+^ species in the water, favoring the reaction with CO_2_. Consequently, the pressure tended to decrease quickly in the first few hours. Using both the Rietveld data (from 8.9% to 5.1%) and the XRD pattern, in LF_2, the decrease in periclase occurred but there was no evidence of crystalline magnesite formation in the XRD pattern (Figure 2b). Amorphous magnesite may be formed but this evidence could not be confirmed because the composition of the amorphous phase is unknown. Both LF_2 and AOD_1 showed the formation of calcite and aragonite, with LF_2 confirmed using the IR spectra at 1470 cm^−1^ (see the Supplementary Materials, Figure S5). In addition, these slags contained a low percentage of iron minerals, favoring the carbonation process. In fact, if the tests occur in an oxidative environment (as in this work), the Fe-minerals are oxidized, forming a hematite (Fe_2_O_3_) surface layer, which has a very low solubility [45] and obstructs the CO_2_–slurry interaction, slowing the reaction kinetics and decreasing the ionic diffusivity [43,46]. On the other hand, in all EAF samples, the presence of Fe phases in higher concentrations hindered the slurry preparation, as these Fe phases are insoluble in water, leading to slurry separation into two phases after a short period. As a result, the slurry lost its homogeneity, making the insertion process into the barrel more difficult. The EAF slags exhibited different sequestration potentials. Among the EAF slags, EAF_3 showed the best performance due to its lower Fe phase quantity and higher amorphous silica, which is soluble at high pH [47]. This allowed for better dissolution of calcium ions and slurry homogeneity. The Rietveld method indicated that the amorphous phase decreased in carbonated products (from 82.7% to 62.3% in weight), and it may contain some reactive species that cannot be identified. EAF_2 with or without SiO_2_ exhibited different results: in the first case, calcite was formed, whereas, in the second case, calcite monohydrate (CaCO_3_*H_2_O) was formed.

**Figure A1:**
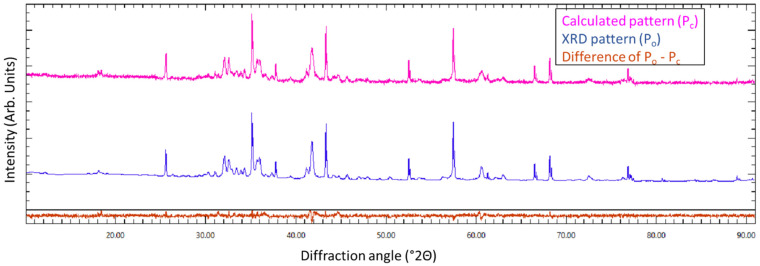
EAF_1 PROFEX profile for the Rietveld calculation.

**Figure A2:**
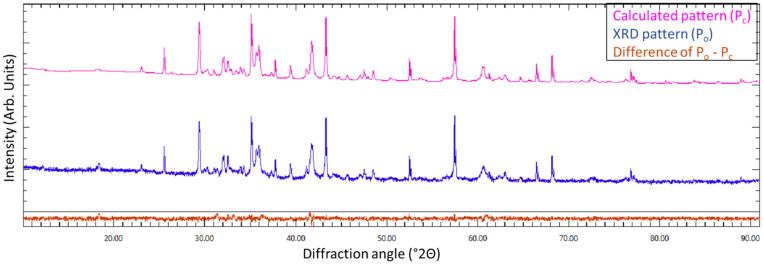
Carbonated EAF_1 PROFEX profile for the Rietveld calculation.

In the EAF_2 + SiO_2_ sample, the amorphous phase remained unaffected, as observed in the Rietveld data (with a weight range of 71.1% to 73.5%, see Table A3), but the pH of the slurry changed: without silica, the value was 10 and increased to 12 when silica was added. This experimental evidence suggests that SiO_2_ contributed to favoring the carbonation process and it also contributed to homogenizing and increasing the solubility of the slag at high pH. The EAF_1 sample displayed good sequestration capabilities, with the dicalcium silicate reducing accordingly (from 28.5% to 21.2%), as seen in other EAF slags. It contained the highest percentage of CaO relative to other EAF slags, but the decantation of the slurry (the inserted mass of the powder was minor at 30 g, while the mass inserted for the other slags was about 30 g) and the iron interferences probably made the gas–slurry interaction difficult. For EAF_1 and its related product, the PROFEX profile for the Rietveld quantification is shown in Figure A1 and Figure A2. The figure reports the collected patterns, the pattern calculated by the software, and the difference between these two patterns. If the difference is a line, it means that the calculated and collected patterns match and the Rietveld refinement is good. EAF_1 and EAF_2 had similar pressure trends (Figure 3) in the first 2 h, probably due to the rapid leaching of calcium ions; the change of the trend may have been due to the decantation of iron components.

CKD showed the worst pressure trend (Figure 3), probably due to the presence of calcite as the primary phase, which has low solubility in water and does not react with the gas. Despite this, CKD demonstrated a strong sequestration capacity, probably due to the calcium oxide present not only in crystalline form but probably also in the amorphous phase due to the pick-up point of CKD [6]. In fact, the amorphous phase decreased significantly (from approximately 6.9% to around <1% in weight, as shown in Table A2) in the carbonated product. Unfortunately, the chemical composition of the amorphous phase could not be accurately identified. However, the FT-IR analysis revealed the probable formation of a Ca-containing silicate-rich gel at 1060 cm^−1^ (which was not identified by the XRD analysis) [48] and confirmed the presence of calcite in the region of 800–1500 cm^−1^ (refer to Figure S7 in the Supplementary Materials).

Based on the results obtained, it was possible to estimate the theoretical sequestration potential of the starting materials by assuming a complete reaction of all reactive species. The Rietveld calculation and the average value show that AOD/LF slags and EAF slags could sequester 105 g CO_2_/kg of slag and 135 g CO_2_/kg of slag if the larnite phase reacted completely. Although this estimation did not consider other reactive Ca species, such as merwinite or gehlenite, the real capability of slags could be better, especially if the average %CaO between AOD/LF and EAF, as shown in Table 1, was taken into account. This could increase the sequestration potential to 313 g CO_2_/kg slag and 200 g CO_2_/kg slag, respectively, and these data are consistent with the literature results [10,49]. In both starting materials, the amorphous phase may play a relevant role in the process, but its identification and, consequently, quantification of chemical species in this part was not possible with the instruments available. Moreover, estimating the theoretical CO_2_ sequestration capacity of CKD was more challenging. However, based on the LOI and %CaO shown in Table 1, the %CaO that was free could be 7%, and the potential sequestration of CKD could be 55 g CO_2_/kg of CKD. It is worth noting that this value is underestimated compared with the values reported in the literature [50,51].

It is important to note that the reported values are theoretical, and the exact experimental sequestration capacity of these materials is still unknown. This is because the CO_2_ was not injected continuously, but in a specific quantity during the screening.

**Figure 5:**
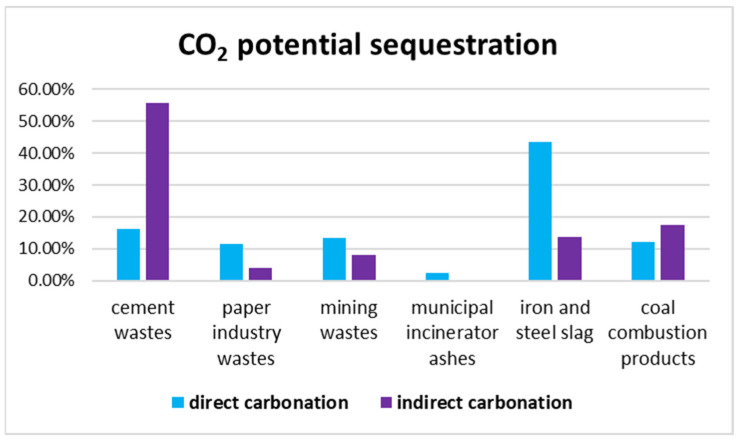
Potential sequestration of industrial wastes using a direct and indirect carbonation reaction in global CO_2_ reduction.

The industrial sequestration potential of CKD and steel slag was estimated based on the obtained and theoretical values of the captured CO_2_. CKD production typically results in 54–200 kg of waste per ton of cement clinker [51]. Assuming a production of 1 ton of CKD, the potential CO_2_ capture ranges from 48 kg to 55 kg, considering the obtained and theoretical values, respectively. On the other hand, steel slag contains several free Ca-phases that can react with CO_2_, and it was estimated that the proposed method could sequester 124–240 kg CO_2_ per ton of slag based on the average experimental and theoretical values of sequestered carbon dioxide calculated previously, whereas they can sequester 513 kg CO_2_ for 1 ton of slag if the average %CaO was considered. The direct carbonation process of steel slag can be more efficient than CKD because of the higher availability of free Ca phases. However, the CKD could be more effective in an indirect route that optimizes Ca leaching through a solvent, as shown also in Figure 5 [49,52,53]. The indirect route was carried out in two separate steps: the leaching of Ca^2+^ and Mg^2+^ ions from wastes using chemical solvents (sulfuric acid, hydrochloric acid, sodium hydroxide, ammonium hydroxide, etc.) and the reaction between lixiviate material and CO_2_. The choice of extraction conditions is fundamental in this method and, in general, acid solvents are used for Ca^2+^ ions extraction, whereas basic solvents are used for Mg^2+^ ions [1,4,54].

Federacciai, which is the Italian consortium of steel plants, reported the production of 461,318 tons of white slags and 1,508,622 tons of EAF slags in Lombardy in 2021 [55]. Based on the potential sequestration results of these starting materials, the proposed method could sequester about 220,000–470,000 tons of CO_2_ per year. However, the mineral storage of steel slag cannot be the sole solution for CO_2_ sequestration [56], and other methods need to be explored to achieve the net zero carbon emission goal by 2050, as established by COP26. Figure 5 [49] highlights the relevance of steel slag in the carbonation process, as it accounts for 43.5% of the 310 Mt CO_2_ sequestered globally through direct carbonation.

### SEM Analysis

**Figure 6:**
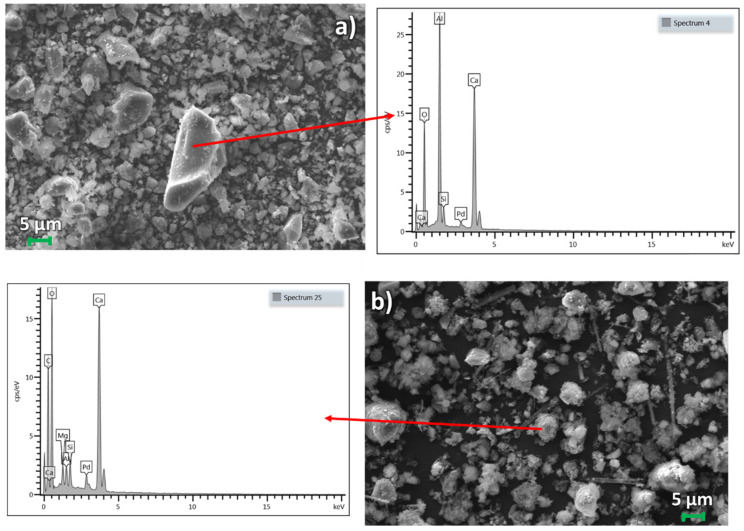

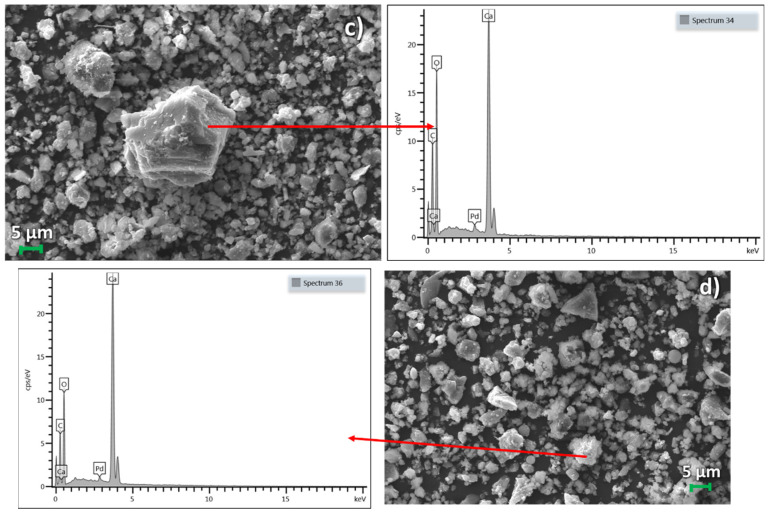
SEM images of the AOD_1 slag (**a**), AOD_1_carbonated (**b**), CKD (**c**), and CKD_carbonated (**d**).

We conducted SEM analyses on the best-performing steel slag (EAF_3 + SiO_2_, AOD_1, and LF_2) and CKD. Only AOD_1 slag showed visible differences between the starting material and the carbonated product. The steel slag before carbonation was homogeneous and contained different crystals of Ca_2_SiO_4_, gehlenite (Ca_2_Al(AlSi)O_7_), or garnet, as shown in Figure 6a. In AOD_1, calcite was irregularly crystallized, growing on Ca-phase crystal surfaces, as shown in Figure 6b. The long needles present in the AOD_1 carbonated sample were attributed to the hydration of calcium silicate, where calcium carbonate particles formed on the surface, in accordance with the chemical analysis (Figure S1 in the Supplementary Materials) [57]. In LF_2, CaCO_3_ was present as calcite and aragonite, which were long needles (see the Supplementary Materials, Figure S1), as confirmed by the XRD pattern (Figure 2b) [58,59,60,61,62]. In EAF_3 + SiO_2_, the starting material was not homogeneous, with three phases being distinguished: amorphous silica, which was present as small circular particles and a pile, and slag as crystals (see the Supplementary Materials, Figure S2). After carbonation, the sample was more homogeneous, with visible calcite crystals growing on Ca_2_SiO_4_ particles (see the Supplementary Materials, Figure S3). The CKD showed calcite as the main phase, with interesting small crystals of CaCO_3_ growing differently from the initial crystal of calcite due to the absence of control of particle growth of the interested product (Figure 6c,d) [63].

## 4. Conclusions

This study proposed a novel method of accelerated carbonation in a pressurized reactor for the by-products of Brescia province steel plants. This method allowed for the efficient and rapid quantification of the sequestration capacities of these collected materials, with the aim of identifying the most promising candidates for use as CO_2_ sponges in the carbonation process. The results indicated that AOD/LF slags could capture 105 g CO_2_/kg of slag and EAF slag could capture 135 g CO_2_/kg of slag if all calcium silicate phases reacted, and further optimization of the process and reactivity of other phases can improve these results. The potential of CKD was underestimated at 55 g CO_2_/kg CKD, likely due to unsuitable reaction conditions.

This paper reports the initial screening results, with further research aimed at finding the optimal reaction conditions in terms of the liquid–solid ratio, pressure, granulometry, and temperature to enhance the carbonation process. The carbonated products could potentially be used as a constituent in cement or as an addition to concrete, following validation of mechanical, hydraulic, and durability tests, as well as an assessment of their environmental impact. Additionally, if the carbonated materials are inert, they could be used as filler in concrete. However, when reusing carbonated steel slags in cementitious materials, the presence of iron oxide in different forms must be considered, as it can influence the morphology and cementitious properties of the final product [64]. It is therefore crucial to develop an efficient strategy to reduce or remove these Fe phases, particularly the magnetic components, which can increase the hardness of the slag and make grinding and crushing activities difficult and energy-consuming.

## Figures and Tables

**Table 1:** Chemical compositions of the starting materials obtained via XRF analysis (LOQ—limit of quantification).

		AOD_1	LF_2	EAF_1	EAF_2	EAF_3 + SiO_2_	CKD
LOI	%	−0.7	5.7	−3.3	−3.0	1.1	29.9
SiO_2_	%	23 ± 1	17.4 ± 0.6	10.9 ± 0.5	12.4 ± 0.5	27 ± 1	12.9 ± 0.5
Al_2_O_3_	%	10.8 ± 0.5	10.4 ± 0.5	6.7 ± 0.4	7.9 ± 0.4	2.2 ± 0.2	2.5 ± 0.2
Fe_2_O_3_	%	5.2 ± 0.4	12.8 ± 0.5	38 ± 1	44 ± 1	30 ± 1	0.2 ± 0.1
CaO	%	49 ± 1	36 ± 1	30 ± 1	23 ± 1	25 ± 1	45 ± 1
MgO	%	8.5 ± 0.4	11.6 ± 0.5	6.0 ± 0.4	6.2 ± 0.4	6.2 ± 0.4	0.5 ± 0.1
SO_3_	%	0.2 ± 0.1	1.4 ± 0.2	0.3 ± 0.1	0.4 ± 0.1	0.2 ± 0.1	4.9 ± 0.4
K_2_O	%	<LOQ	0.5 ± 0.1	<LOQ	<LOQ	0.4 ± 0.1	1.0 ± 0.2
Na_2_O	%	0.8 ± 0.1	0.3 ± 0.1	<LOQ	0.1 ± 0.1	0.3 ± 0.1	2.7 ± 0.2
TiO_2_	%	0.3 ± 0.1	1.2 ± 0.2	0.2 ± 0.1	0.6 ± 0.1	0.2 ± 0.1	<LOQ
P_2_O_5_	%	<LOQ	<LOQ	0.3 ± 0.1	0.3 ± 0.1	0.3 ± 0.1	<LOQ
Mn_2_O_3_	%	1.0 ± 0.1	1.6 ± 0.2	7.0 ± 0.4	7.0 ± 0.4	5.9 ± 0.4	<LOQ

**Table 2:** Trends of the reaction parameters in carbonation test for investigated samples (argon oxygen decarburization (AOD_1) slag, ladle furnace (LF_2) slag, electric arc furnace (EAF_1, EAF_2, EAF_3) slag, and cement kiln dust (CKD)).

	AOD_1	LF_2	EAF_1	EAF_2	EAF_2 + SiO_2_	EAF_3 + SiO_2_	CKD
**pH_i_**	13	13	12	10	12	13	12
**p_i_ (bar)**	15.1	15.1	14.9	15.0	15.0	15.0	15.0
**pH_f_**	9	9	9	8	8.5	10	8
**p_f_ (bar)**	2.2	4.0	6.0	7.2	7.1	5.5	7.6
**m_i_ CO_2_ (g)**	2.53	2.59	2.44	2.59	2.69	2.69	2.57

## Data Availability

The data is available in the manuscript and Supplementary Materials.

## Supplementary Materials

The following supporting information can be downloaded from https://www.mdpi.com/article/10.3390/ma16114055/s1: Figure S1: SEM images of (a) LF_2, (b) carbonated LF_2, and (c) carbonated AOD_1; Figure S2: SEM images of (a) EAF_3 + SiO_2_ and (b) EAF_3 + SiO_2_ carbonated products; Figure S3: SEM images of the calcite present in EAF_3 + SiO_2__carbonated at (a) 5000× and (b) 15,000×; Figure S4: IR spectrum of AOD_1 and AOD_1_carbonated; Figure S5: IR spectra of LF_2 and LF_2_carbonated; Figure S6: IR spectra of EAF_3 + SiO_2_ and EAF_3 + SiO_2__carbonated; Figure S7: IR spectra of CKD and CKD_carbonated.

## Appendix A

In this section, information on the mineralogical phases, Rietveld data, and PROFEX profile are reported.

**Table A1:** Mineralogical phases identification.

Mineralogical Phases	Chemical Formula	Reference Code	Industrial Waste
Albite	NaAlSi_3_O_8_	00-009-0466	CKD
Aragonite	CaCO_3_	00-041-1475	AOD_1, LF_2
Bredigite	Ca_1_._7_Mg_0_._3_SiO_4_	00-035-0260	AOD_1
Brownmillerite	Ca_2_(Al,Fe)_2_O_5_	00-030-0226	EAF_1
Brucite	Mg(OH)_2_	01-074-2220	LF_2
Calcite	CaCO_3_	01-080-0941	AOD_1, LF_2
Calcite	CaCO_3_	00-005-0586	EAF_1, CKD
Calcite	CaCO_3_	01-072-1937	EAF_2, EAF_3 + SiO_2_
Calcite monohydrate	CaCO_3_*H_2_O	01-084-0049	EAF_2 + SiO_2_
Calcium oxide	CaO	01-082-1690	CKD
Calcium silicate	Ca_2_SiO_4_	00-031-0297	AOD_1
Calcium silicate	Ca_2_SiO_4_	00-036-0642	AOD_1
Dolomite	CaMg(CO_3_)_2_	01-075-1710	AOD_1
Garnet	Ca_1_._92_Fe_3_._08_O_12_Si_3_	01-082-1552	AOD_1
Gehlenite	Ca_2_Al_2_SiO_7_	00-020-0199	AOD_1, LF_2
Gehlenite	Ca_2_Al_2_SiO_7_	00-009-0216	EAF_2, EAF_2 + SiO_2_
Hydrogarnet	Ca_3_Al_2_(H_4_O_4_)_3_	01-084-1353	LF_2
Larnite	Ca_2_SiO_4_	00-033-0302	LF_2, EAF_1
Larnite	Ca_2_SiO_4_	00-029-0369	EAF_2, EAF_2 + SiO_2_
Larnite	Ca_2_SiO_4_	00-020-0237	EAF_3 + SiO_2_
Maghemite	Fe_2_O_3_	00-025-1402	EAF_1
Magnetite	Fe_3_O_4_	01-079-0418	EAF_1, EAF_2, EAF_2 + SiO_2_
Magnetite	Fe_3_O_4_	01-072-2303	EAF_3 + SiO_2_
Mayenite	Ca_12_Al_14_O_33_	00-009-0413	AOD_1
Olivine	Ca_2_SiO_4_	01-080-0941	LF_2
Periclase	MgO	01-036-0678	AOD_1
Portlandite	Ca(OH)_2_	00-044-1481	AOD_1,EAF_1
Quartz	SiO_2_	00-046-1045	CKD
Quartz	SiO_2_	01-079-19-06	LF_2
Srebrodolskite	Ca_2_Fe_2_O_5_	01-074-1860	EAF_2, EAF_2 + SiO_2_
Wuestite	FeO	01-077-2355	EAF_1
Wuestite	Fe_0_._974_O	01-073-2143	LF_2, EAF_2, EAF_2 + SiO_2_
Yoshiokaite	Ca_2_(Al,Si)_2_O_4_	00-046-1336	EAF_2, EAF_2 + SiO_2_

**Table A2:** Rietveld data of the AOD_1, LF_2, CKD, and related products.

		AOD_1	Carbonated AOD_1	LF_2	Carbonated LF2	CKD	Carbonated CKD
Albite	%					<1	<1
Garnet	%	3.9	3.6				
Hydrogarnet	%			8.8	14.3		
Mayenite	%	12	2.8				
Bredigite	%	2.2	2.2				
Brucite	%			<1	<1		
Dolomite	%	3.7	3	1.45	<1		
Gehlenite	%	<1	<1	8.1	5.4		
Periclase	%	5.6	5.2	8.95	5.1		
Portlandite	%	1	< 1				
Calcium silicate	%	20.7	12.3				
Quartz	%			1.3	2.2	6	5.6
Merwinite	%			4.6	0.5		
Wuestite	%			2.9	<1		
Larnite	%			13.1	8.3		
Olivine	%			15.6	17.7		
Lime	%					1.1	<1
Calcite	%	2.7	16.7	3.2	8.8	86	94
Aragonite	%	<1	1	<1	8.7		
Amorphous	%	49.0	54.0	31.4	29.5	6.9	<1

**Table A3:** Rietlveld data of the EAF_1, EAF_2, EAF_2 + SiO_2_, EAF_3 + SiO_2_, and related carbonated products.

		EAF_1	Carbonated EAF_1	EAF_2	Carbonated EAF_2	EAF_2 + SiO2	Carbonated EAF_2 + SiO2	EAF_3 + SiO2	Carbonated EAF_3 + SiO2
Browmillerite	%	7.4	9.31						
Portlandite	%	1	<1						
Larnite	%	28.5	21.2	10.3	1.3	6.1	<1	7.2	6.7
Maghemite	%	6.9	7.8						
Wuestite	%	11.3	11.7	18.5	9.3	9.3	7.3		
Gehlenite	%			11.5	8.8	7.4	6.2		
Magnetite	%	2.6	3.1	3	5.7	3.2	2.1	9.2	16.6
Yoshiokaite	%			<1	3.6	<1	<1		
Srebrodolskite	%			4.4	3.7	2.6	< 1		
Calcite	%	<1	16.7	<1	7.5			<1	14
Monohydrate calcite	%					<1	9.2		
Amorphous	%	41.8	29.8	52.0	60.2	71.1	73.5	82.7	62.3

## References

[1] Liu W., Teng L., Rohani S., Qin Z., Zhao B., Xu C.C., Ren S., Liu Q., Liang B. (2021). CO_2_ Mineral Carbonation Using Industrial Solid Wastes: A Review of Recent Developments. Chem. Eng. J..

[2] Jim Skea P.R.S. (2022). Climate Change 2022.

[3] European Union (2009). Direttiva 2009/31/CE del Parlamento europeo e del Consiglio del 23 Aprile 2009.

[4] Yadav S., Mehra A. (2021). A Review on Ex Situ Mineral Carbonation. Environ. Sci. Pollut. Res..

[5] Wang D., Xiao J., Duan Z. (2022). Strategies to Accelerate CO_2_ Sequestration of Cement-Based Materials and Their Application Prospects. Constr. Build. Mater..

[6] Sorrentino G.P., Zanoletti A., Ducoli S., Zacco A., Iora P., Invernizzi C.M., Di Marcoberardino G., Depero L.E., Bontempi E. (2023). Accelerated and Natural Carbonation of a Municipal Solid Waste Incineration (MSWI) Fly Ash Mixture: Basic Strategies for Higher Carbon Dioxide Sequestration and Reliable Mass Quantification. Environ. Res..

[7] Zhang P., Lewis J.B., Klein-BenDavid O., Garrabrants A.C., Delapp R., van der Sloot H.A., Kosson D.S. (2022). The Role of Environmental Conditions on the Carbonation of an Alkali-Activated Cementitious Waste Form. Cem. Concr. Res..

[8] Polettini A., Pomi R., Stramazzo A. (2016). Carbon Sequestration through Accelerated Carbonation of BOF Slag: Influence of Particle Size Characteristics. Chem. Eng. J..

[9] Pan S.-Y., Chang E.E., Chiang P.-C. (2012). CO_2_ Capture by Accelerated Carbonation of Alkaline Wastes: A Review on Its Principles and Applications. Aerosol Air Qual. Res..

[10] Baciocchi R., Costa G., Di Gianfilippo M., Polettini A., Pomi R., Stramazzo A. (2015). Thin-Film versus Slurry-Phase Carbonation of Steel Slag: CO_2_ Uptake and Effects on Mineralogy. J. Hazard. Mater..

[11] Huntzinger D.N., Gierke J.S., Kawatra S.K., Eisele T.C., Sutter L.L. (2009). Carbon Dioxide Sequestration in Cement Kiln Dust through Mineral Carbonation. Environ. Sci. Technol..

[12] Neeraj, Yadav S. (2020). Carbon Storage by Mineral Carbonation and Industrial Applications of CO_2_. Mater. Sci. Energy Technol..

[13] Olabi A.G., Wilberforce T., Elsaid K., Sayed E.T., Maghrabie H.M., Abdelkareem M.A. (2022). Large Scale Application of Carbon Capture to Process Industries—A Review. J. Clean. Prod..

[14] Eurofer (2021). European Steel in Figures 2022.

[15] Falsafi M., Fornasiero R. (2022). Explorative Multiple-Case Research on the Scrap-Based Steel Slag Value Chain: Opportunities for Circular Economy. Sustainability.

[16] Bonenfant D., Kharoune L., Sauve´ S., Hausler R., Niquette P., Mimeault M., Kharoune M. (2008). CO_2_ Sequestration Potential of Steel Slags at Ambient Pressure and Temperature. Ind. Eng. Chem. Res..

[17] Huijgen W.J.J., Witkamp G.-J., Comans R.N.J. (2005). Mineral CO_2_ Sequestration by Steel Slag Carbonation. Environ. Sci. Technol..

[18] Huijgen W.J.J., Comans R.N.J. (2006). Carbonation of Steel Slag for CO_2_ Sequestration: Leaching of Products and Reaction Mechanisms. Environ. Sci. Technol..

[19] Fang Y., Zhang Y., Zhang M., Zhao M., Wang Q. (2022). Carbonation Curing of Alkaline Industrial Waste for Binders: Comparison of Different Wastes. Mag. Concr. Res..

[20] Hou G., Yan Z., Sun J., Naguib H.M., Lu B., Zhang Z. (2021). Microstructure and Mechanical Properties of CO_2_-Cured Steel Slag Brick in Pilot-Scale. Constr. Build. Mater..

[21] Ortega I., Faik A., Gil A., Rodríguez-Aseguinolaza J., D’Aguanno B. (2015). Thermo-Physical Properties of a Steel-Making by-Product to Be Used as Thermal Energy Storage Material in a Packed-Bed System. Energy Procedia.

[22] Gadikota G., Park A.A. (2015). Accelerated Carbonation of Ca- and Mg-Bearing Minerals and Industrial Wastes Using CO_2_. Carbon: Dioxide Utilisation.

[23] Polettini A., Pomi R., Stramazzo A. (2016). CO_2_ Sequestration through Aqueous Accelerated Carbonation of BOF Slag: A Factorial Study of Parameters Effects. J. Environ. Manag..

[24] Song Q., Guo M.-Z., Wang L., Ling T.-C. (2021). Use of Steel Slag as Sustainable Construction Materials: A Review of Accelerated Carbonation Treatment. Resour. Conserv. Recycl..

[25] Al-Bakri A.Y., Ahmed H.M., Hefni M.A. (2022). Cement Kiln Dust (CKD): Potential Beneficial Applications and Eco-Sustainable Solutions. Sustainability.

[26] Li L., Ling T.-C., Pan S.-Y. (2022). Environmental Benefit Assessment of Steel Slag Utilization and Carbonation: A Systematic Review. Sci. Total Environ..

[27] Anastasiou E.K., Liapis A., Papachristoforou M. (2017). Life Cycle Assessment of Concrete Products for Special Applications Containing EAF Slag. Procedia Environ. Sci..

[28] Abdel-Ghani N.T., El-Sayed H.A., El-Habak A.A. (2018). Utilization of By-Pass Cement Kiln Dust and Air-Cooled Blast-Furnace Steel Slag in the Production of Some “Green” Cement Products. HBRC J..

[29] Sharma D., Goyal S. (2018). Accelerated Carbonation Curing of Cement Mortars Containing Cement Kiln Dust: An Effective Way of CO_2_ Sequestration and Carbon Footprint Reduction. J. Clean. Prod..

[30] Huntzinger D.N., Gierke J.S., Sutter L.L., Kawatra S.K., Eisele T.C. (2009). Mineral Carbonation for Carbon Sequestration in Cement Kiln Dust from Waste Piles. J. Hazard. Mater..

[31] Federacciai (2012). La Valorizzazione degli Aggregati di Origine Siderurgica.

[32] Baciocchi R., Costa G., di Bartolomeo E., Polettini A., Pomi R. (2010). Carbonation of Stainless Steel Slag as a Process for CO_2_ Storage and Slag Valorization. Waste Biomass Valoriz..

[33] Chiang P.-C., Pan S.-Y. (2017). Carbon Dioxide Mineralization and Utilization.

[34] Doebelin N., Kleeberg R. (2015). Profex: A Graphical User Interface for the Rietveld Refinement Program BGMN. J. Appl. Cryst..

[35] Assi A., Federici S., Bilo F., Zacco A., Depero L.E., Bontempi E. (2019). Increased Sustainability of Carbon Dioxide Mineral Sequestration by a Technology Involving Fly Ash Stabilization. Materials.

[36] Brand A.S., Fanijo E.O. (2020). A Review of the Influence of Steel Furnace Slag Type on the Properties of Cementitious Composites. Appl. Sci..

[37] Alanyalı H., Çöl M., Yılmaz M., Karagöz Ş. (2006). Application of Magnetic Separation to Steelmaking Slags for Reclamation. Waste Manag..

[38] Abdelzaher M., Hamouda A., El-Kattan I., Baher A. (2022). Laboratory Study for Accelerating the CKD Mineral Carbonation. Egypt. J. Chem..

[39] Capeletti L.B., Zimnoch J.H. (2016). Fourier Transform Infrared and Raman Characterization of Silica-Based Materials. Applications of Molecular Spectroscopy to Current Research in the Chemical and Biological Sciences.

[40] Baciocchi R., Costa G., Polettini A., Pomi R. (2009). Influence of Particle Size on the Carbonation of Stainless Steel Slag for CO_2_ Storage. Energy Procedia.

[41] Xu B., Yi Y. (2019). Soft Clay Stabilization Using Ladle Slag-Ground Granulated Blastfurnace Slag Blend. Appl. Clay Sci..

[42] Chai Y.E., Miller Q.R.S., Schaef H.T., Barpaga D., Bakhshoodeh R., Bodor M., Van Gerven T., Santos R.M. (2021). Pressurized in Situ X-Ray Diffraction Insights into Super/Subcritical Carbonation Reaction Pathways of Steelmaking Slags and Constituent Silicate Minerals. J. Supercrit. Fluids.

[43] Bodor M., Santos R.M., Kriskova L., Elsen J., Vlad M., Van Gerven T. (2013). Susceptibility of Mineral Phases of Steel Slags towards Carbonation: Mineralogical, Morphological and Chemical Assessment. Eur. J. Mineral..

[44] Johnson D.C., Macleod C.L., Carey P.J., Hills C.D. (2003). Solidification of Stainless Steel Slag by Accelerated Carbonation. Environ. Technol..

[45] Jang J.-H., Dempsey B.A., Burgos W.D. (2007). Solubility of Hematite Revisited: Effects of Hydration. Environ. Sci. Technol..

[46] Baciocchi R., Costa G., Zingaretti D., Cazzotti M., Werner M., Polettini A., Pomi R., Falasca M. (2010). Studio Sulle Potenzialità della Carbonatazione di Minerali e Residui Industriali per lo Stoccaggio di Anidride Carbonica Prodotta da Impianti di Piccola/Media Taglia.

[47] Alexander G.B., Heston W.M., Iler R.K. (1954). The Solubility of Amorphous Silica in Water. J. Phys. Chem..

[48] Yu P., Kirkpatrick R.J., Poe B., McMillan P.F., Cong X. (2004). Structure of Calcium Silicate Hydrate (C-S-H): Near-, Mid-, and Far-Infrared Spectroscopy. J. Am. Ceram. Soc..

[49] Pan S.-Y., Chen Y.-H., Fan L.-S., Kim H., Gao X., Ling T.-C., Chiang P.-C., Pei S.-L., Gu G. (2020). CO_2_ Mineralization and Utilization by Alkaline Solid Wastes for Potential Carbon Reduction. Nat. Sustain..

[50] Gunning P.J., Hills C.D., Carey P.J. (2010). Accelerated Carbonation Treatment of Industrial Wastes. Waste Manag..

[51] Pedraza J.I., Suarez L.A., Martinez L.A., Rojas N.Y., Tobon J.I., Ramirez J.H., Zea H.R., Caceres A.A. Carbon Capture and Utilization by Mineral Carbonation with CKD in Aqueous Phase: Experimental Stage and Characterization of Carbonated Products. Proceedings of the 7th International Workshop, Advances in Cleaner Production, Academic Work.

[52] Kim M.-J., Pak S.Y., Kim D., Jung S. (2017). Optimum Conditions for Extracting Ca from CKD to Store CO_2_ through Indirect Mineral Carbonation. KSCE J. Civ. Eng..

[53] Irfan M.F., Hossain S.M.Z., Tariq I., Khan N.A., Tawfeeqi A., Goeva A., Wael M. (2020). Modeling and Optimization of Aqueous Mineral Carbonation for Cement Kiln Dust Using Response Surface Methodology Integrated with Box-Behnken and Central Composite Design Approaches. Min. Metall. Explor..

[54] Kunzler C., Alves N., Pereira E., Nienczewski J., Ligabue R., Einloft S., Dullius J. (2011). CO_2_ Storage with Indirect Carbonation Using Industrial Waste. Energy Procedia.

[55] https://Federacciai.It/.

[56] Federacciai (2021). Rapporto di Sostenibilità.

[57] Mo L., Hao Y., Liu Y., Wang F., Deng M. (2019). Preparation of Calcium Carbonate Binders via CO_2_ Activation of Magnesium Slag. Cem. Concr. Res..

[58] Schultz L.N., Andersson M.P., Dalby K.N., Müter D., Okhrimenko D.V., Fordsmand H., Stipp S.L.S. (2013). High Surface Area Calcite. J. Cryst. Growth.

[59] Parker J.E., Thompson S.P., Lennie A.R., Potter J., Tang C.C. (2010). A Study of the Aragonite-Calcite Transformation Using Raman Spectroscopy, Synchrotron Powder Diffraction and Scanning Electron Microscopy. CrystEngComm.

[60] Tao M.-J., Wang Y.-J., Li J.-G., Zeng Y.-N., Liu S.-H., Qin S. (2021). Slurry-Phase Carbonation Reaction Characteristics of AOD Stainless Steel Slag. Processes.

[61] Lee S.M., Lee S.H., Jeong S.K., Youn M.H., Nguyen D.D., Chang S.W., Kim S.S. (2017). Calcium Extraction from Steelmaking Slag and Production of Precipitated Calcium Carbonate from Calcium Oxide for Carbon Dioxide Fixation. J. Ind. Eng. Chem..

[62] Chang E.-E., Chen C.-H., Chen Y.-H., Pan S.-Y., Chiang P.-C. (2011). Performance Evaluation for Carbonation of Steel-Making Slags in a Slurry Reactor. J. Hazard. Mater..

[63] Medas D., Cappai G., De Giudici G., Piredda M., Podda S. (2017). Accelerated Carbonation by Cement Kiln Dust in Aqueous Slurries: Chemical and Mineralogical Investigation. Greenh. Gases Sci. Technol..

[64] Alanyali H., Çöl M., Yilmaz M., Karagöz Ş. (2009). Concrete Produced by Steel-Making Slag (Basic Oxygen Furnace) Addition in Portland Cement. Int. J. Appl. Ceram. Technol..

