# Supplementary material for: Accelerated Direct Carbonation of Steel Slag and Cement Kiln Dust: An Industrial Symbiosis Strategy Applied in the Bergamo–Brescia Area
Source: Materials (Basel). 2023 May 29;16(11):4055. doi: 10.3390/ma16114055 (PMC10254272; doi:10.3390/ma16114055)
Supplement: Supplementary file 1 [file materials-16-04055-s001.zip › materials-2343515-supplementary.pdf]

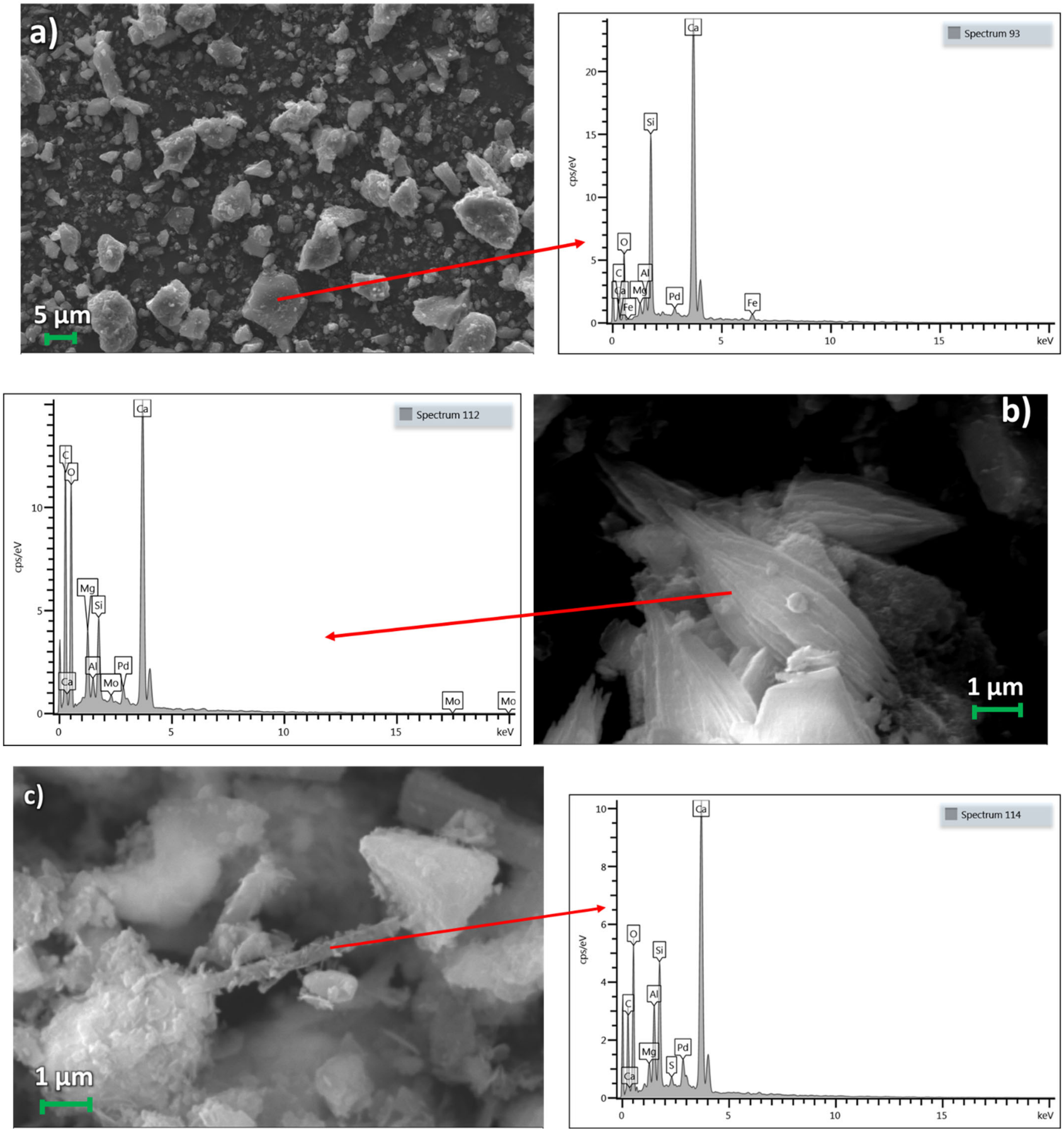

Figure S1. SEM images of LF_2 (a), carbonated LF_2 (b) and c) carbonated AOD_1

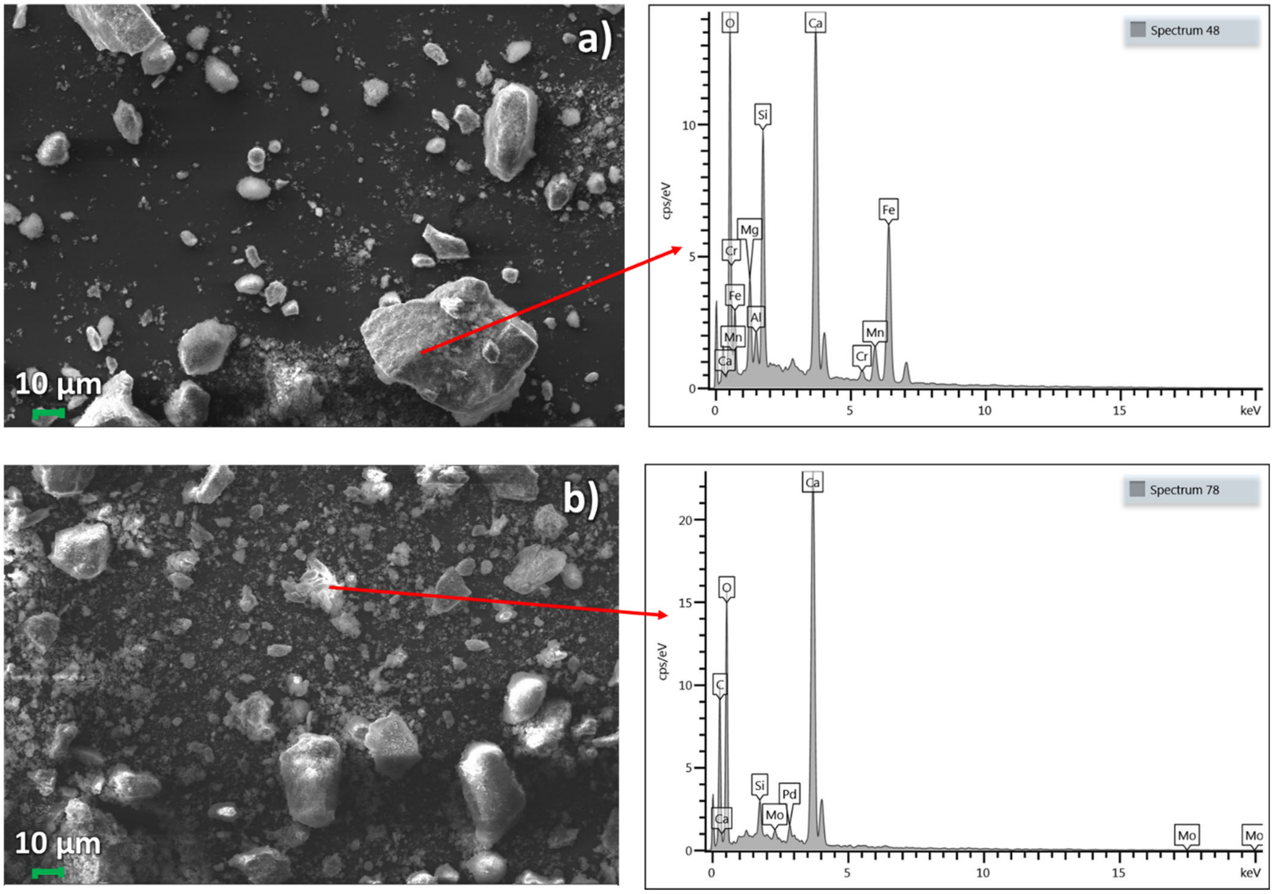

Figure S2. SEM images of EAF_3 + SiO_2 (a) and EAF_3 + SiO_2 carbonated (b) products.

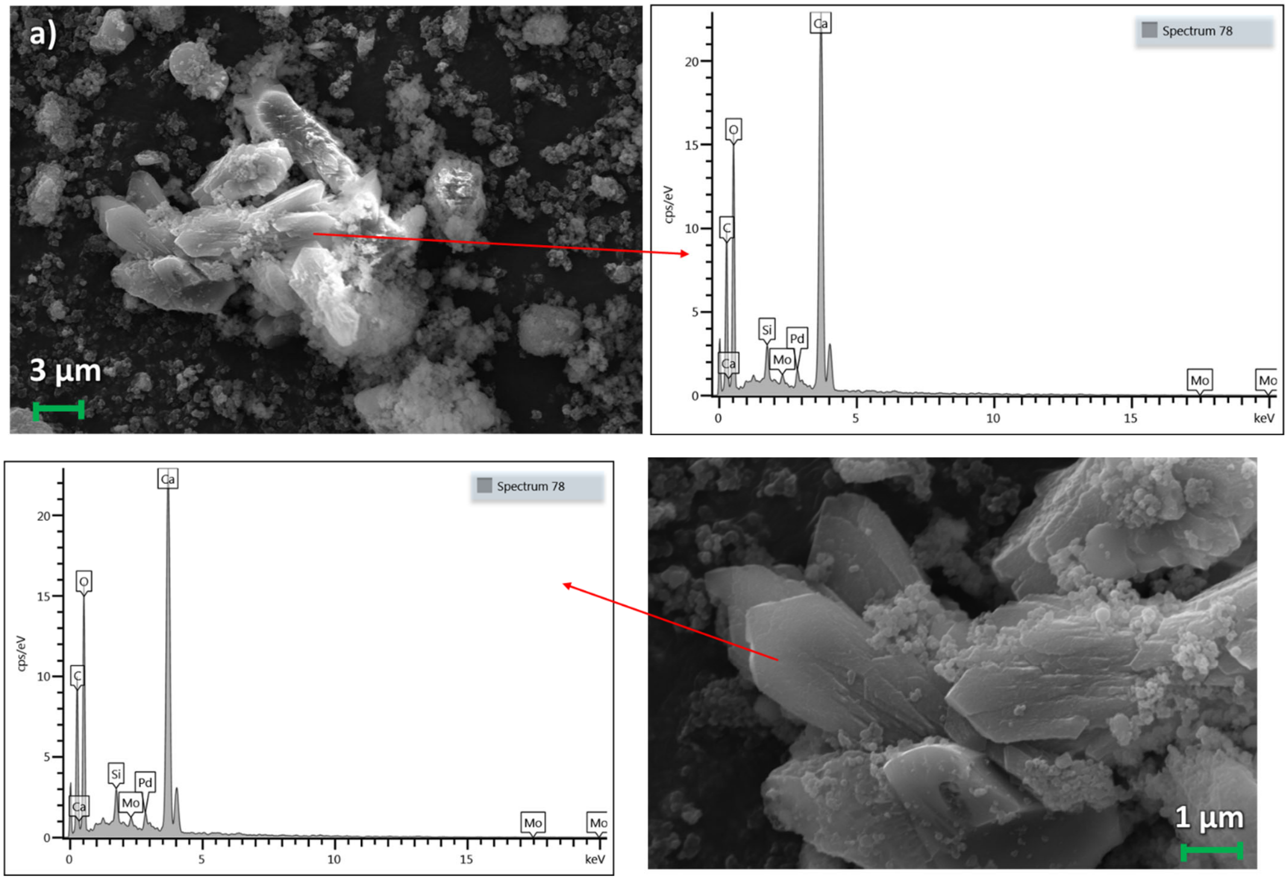

Figure S3. SEM images of calcite present in EAF_3 + SiO_2_carbonated at 5000X (a) and 15000X (b).

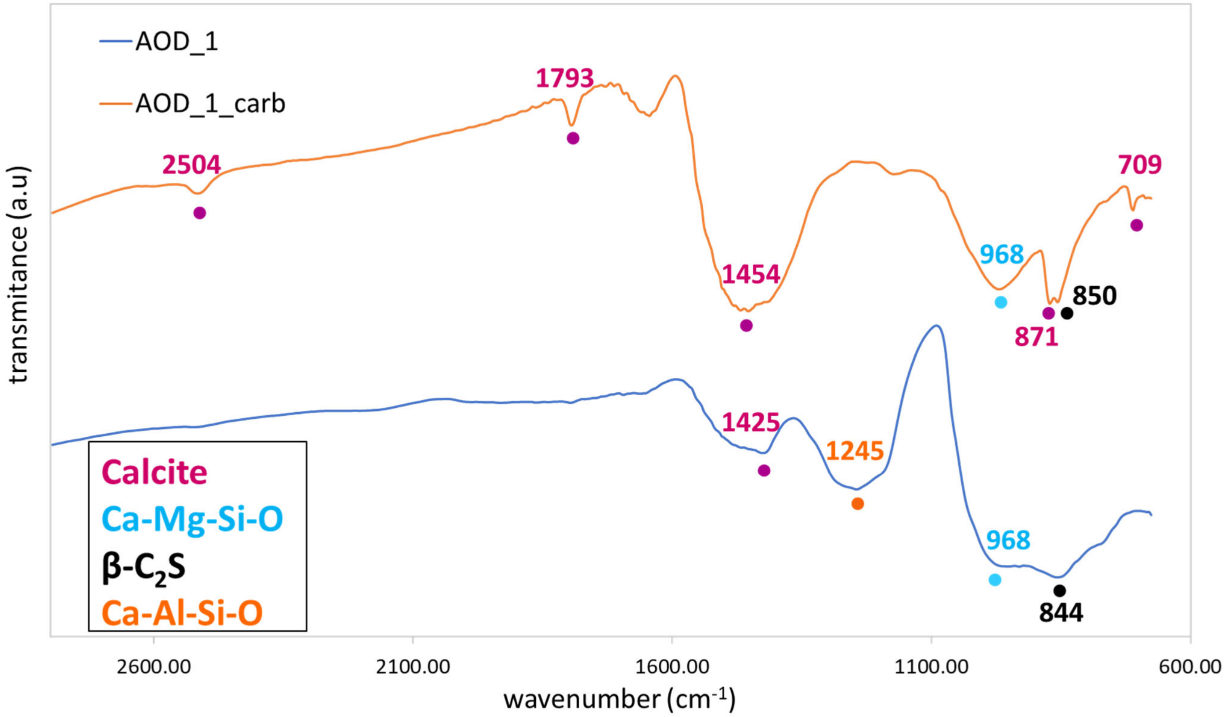

Figure S4. IR spectrum of AOD_1 and AOD_1_carbonated.

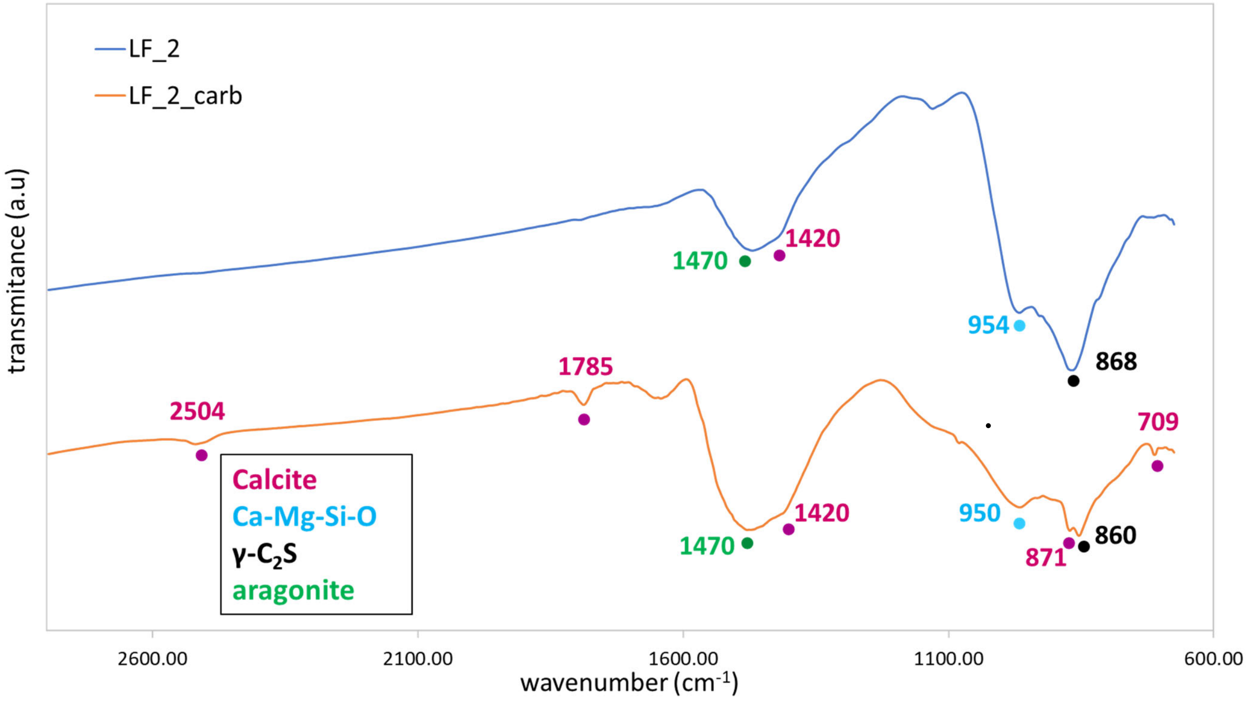

Figure S1. IR spectrum of LF_2 and LF_2_carbonated.

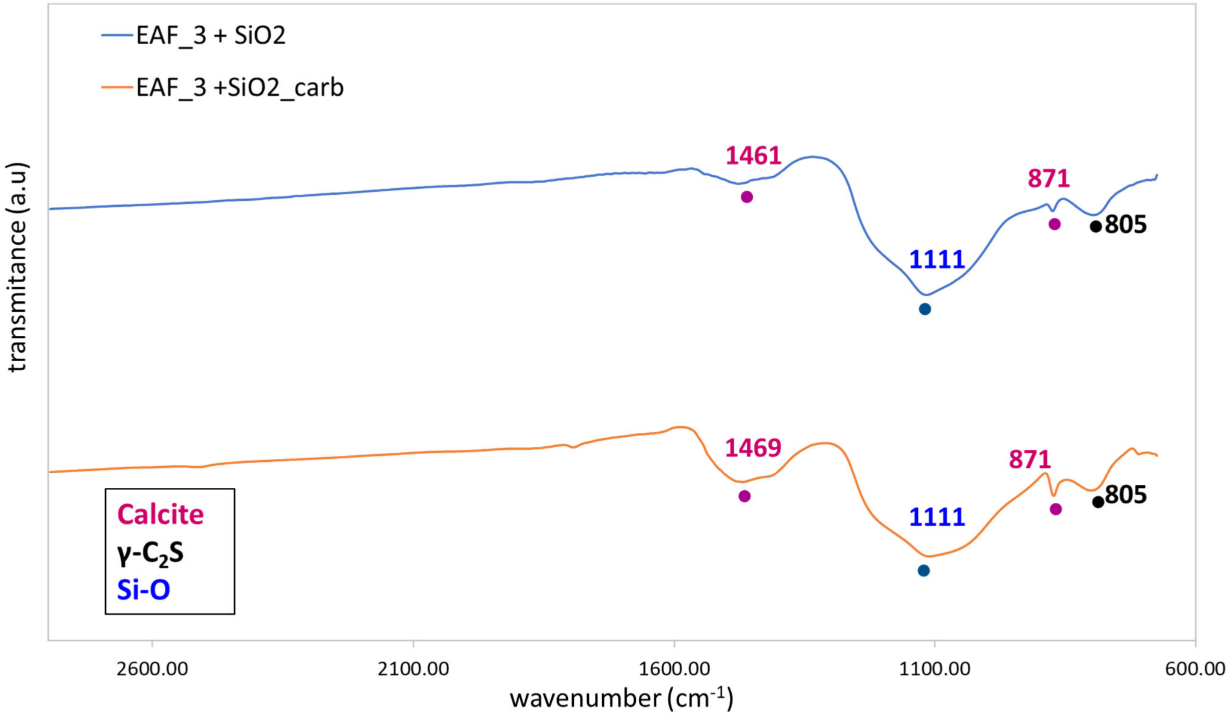

Figure S6. IR spectrum EAF_3 + SiO_2 and EAF_3 + SiO_2_carbonated.

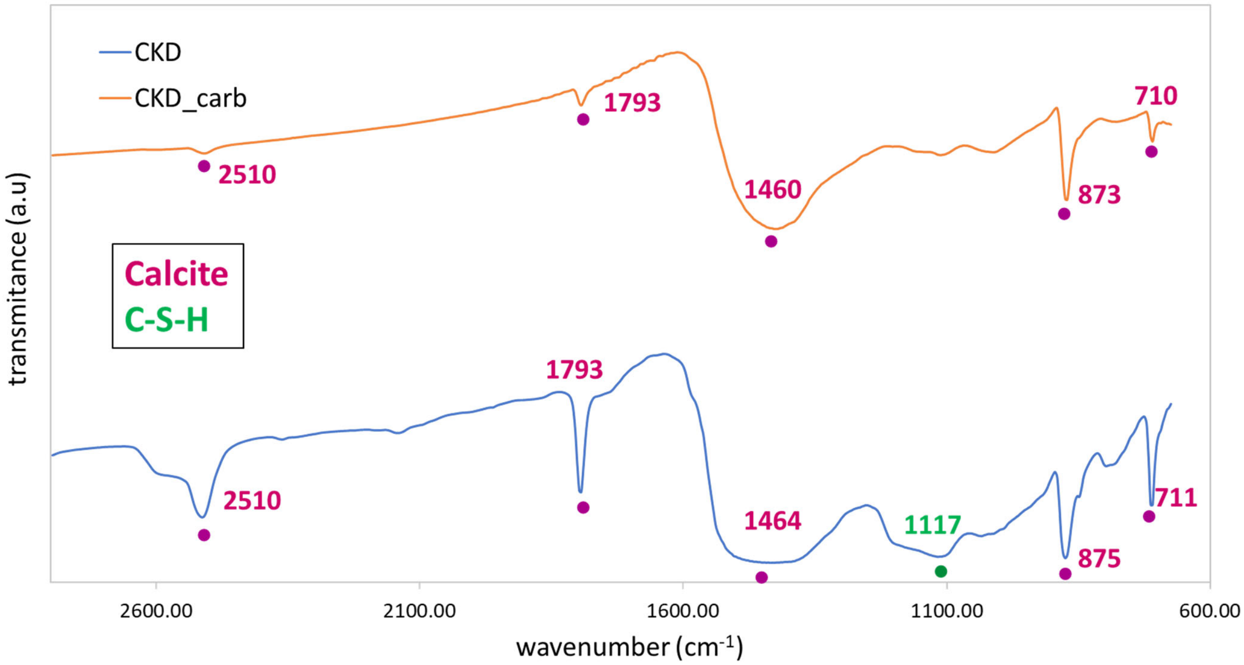

Figure S7. IR spectrum of CKD and CKD_carbonated.
